# Environmental Toxicology in Addressing Public Health Challenges in East Asia

**DOI:** 10.1155/2015/920518

**Published:** 2015-04-29

**Authors:** How-Ran Guo, Zailina Hashim, Shih-Bin Su, Jochen Bundschuh

**Affiliations:** ^1^Department of Environmental and Occupational Health, National Cheng Kung University, Tainan 704, Taiwan; ^2^Department of Occupational and Environmental Medicine, National Cheng Kung University Hospital, Tainan 704, Taiwan; ^3^Department of Environmental and Occupational Health, Universiti Putra Malaysia, 43400 Selangor, Malaysia; ^4^Department of Occupational Medicine, Chi-Mei Medical Center, Tainan 710, Taiwan; ^5^Deputy Vice-Chancellor's Office (Research and Innovation), Research and Innovation and Academic Division and Faculty of Health, Engineering and Sciences, University of Southern Queensland, Toowoomba, QLD 4350, Australia

Environmental toxicology is a multidisciplinary domain of science, which occupies an important niche, overlapping the fields of toxicology, environmental health, and public policy. While studying the adverse health effects of chemical, biological, and physical agents on living organisms in the ecosystems, environmental toxicology focuses on humans and therefore plays an important role in addressing public health challenges. Over the years, environmental toxicology research has been providing tools and scientific evidence to policy-makers and the public in preventing substantially greater environmental degradation, including adverse human health impacts.

In East Asia, with the increasing number of environmental problems, there is a pressing need for immediate health solutions, and environmental toxicology is expected to increasingly play an important role. More fundamental and applied research must be carried out to deal with the public health challenges in terms of environmental problems and to increase the body of scientific knowledge in this field. While environmental toxicology receives increasing attention and shows significant growth, the number of articles published in international journals on environmental toxicology is still relatively limited. As society changes due to introduction of new technologies and the challenge of sustainable development in the face of increased human population, especially in the East Asian region, the role of environmental toxicology in enlightened public health and public policy will become even more important.

Air pollution from both natural and anthropogenic sources now constitutes one of the greatest public health challenges in East Asia at the present time. N. F. Suhaimi and J. Jalaludin conducted a search of journal articles and identified seven studies which used biomarkers to understand the association between air particles exposure and the development of respiratory problems resulting from the damage in the respiratory system. While finding the application of biomarkers in epidemiological studies of health effects caused by air particles in both environmental and occupational health to be inchoate, they concluded that biomarkers can unravel the complexity of the connection between exposure to air particles and respiratory health. N. A. M. N. Rawi et al. carried out a cross-sectional study among Malay children in Selangor to determine the indoor air quality (IAQ) and its association with respiratory health. They measured the levels of particulate matters (PM), volatile organic compounds (VOCs), carbon monoxide (CO), carbon dioxide (CO_2_), air velocity (AV), temperature, and relative humidity and found that abnormality of FVC% among children was associated with the concentrations of PM_2.5_ and CO, while abnormality of FEV1% among children was associated with the concentration of CO. In addition, they found that wheezing was associated with concentrations of PM_2.5_, PM_10_, and CO. M. Watanabe et al. also studied the effects of air pollution on the pulmonary function of school children but targeted at the Asian dust storm (ADS). They identified seven ADS days from 2012 to 2013 in the Shimane Prefecture of Japan and observed a larger change in morning peak expiratory flow (PEF) in children after ADS exposure in 2012 (−8.17 L/min versus −1.17 L/min). Using airborne particles collected on ADS days to stimulate THP-G8 cells, they found interleukin-8 transcriptional activity was also higher when particles collected in 2012 were used. Therefore, they argue that the influence of ADS on pulmonary function of children may be related to interleukin-8 production.

J.-F. Zou et al. studied the health effects of CO but focused on patients suffering from CO poisoning. They conducted a study in the Shandong Province of China and found that a positive Babinski reflex was an independent predictor for delayed neuropsychiatric sequelae (DNS). With a specificity of 88.6% and a negative predictive value of 97.3%, this sign is particularly useful for identifying patients with a low risk of developing DNS. Also targeting neurotoxicity, O. Isildak et al. studied *β*-N-oxalyl-L-*α*,*β*-diaminopropionic acid (ODAP), a toxin that can cause spastic paraplegia. They developed a novel potentiometric sensor based on ionophore (Cd(NH_2_CH_2_CH_2_OCH_2_CH_2_OCH_2_CH_2_NH_2_)Ag_3_(CN)_5_), and detection limit was 2 × 10^−6^ mol L^−1^, with a response time shorter than 6 seconds. The sensor exhibits good operational stability for more than one week in dry conditions at 4–6°C and has a reproducible and stable response during continuous work for more than 10 hours.

Metal and arsenic contamination of water sources also constitute important environmental problems in East Asia and can have severe impacts on humans. A. Waseem et al. reviewed the heavy metal contamination in several areas of Pakistan in recent years, including arsenic, cadmium, lead, chromium, nickel, copper, manganese, iron, and zinc. They found those metals have affected the drinking water quality, ecological environment, and food chain, which poses serious threats to public health. J.-W. Huang et al. focused their study on arsenic and evaluated its association with diabetes mellitus (DM) in Cambodia. They found that drinking water with arsenic levels above 907.25 *μ*g/L and having skin lesions of arsenicosis were associated with a nearly twofold increase in the risk of DM. However, the increases did not reach statistical significance, most likely because of the small case number. The meta-analysis carried out by T.-C. Sung et al. has provided a more comprehensive picture of the association between arsenic exposure and DM. They identified 38 relevant studies, of which the 32 studies on the ingestion route showed a significant association between arsenic exposure and DM. While the heterogeneity among the studies may affect the results, they concluded that ingested arsenic is associated with the development of DM.

The papers of this special issue comprise critical and new areas of research and recent advances on challenging issues in different fields of environmental toxicology. They included contributions from cutting edge researches in environmental toxicology that have been recently conducted in the East Asian region and selected papers from works presented in the third conference organized by the East Asia Chapter of the International Society for Environmental Epidemiology (ISEE) held in Kuala Lumpur in 2012. ISEE's membership is open to environmental epidemiologists and other scientists such as environmental toxicologists, and the biannual conference of the East Asia Chapter provides a forum for discussions, critical reviews, collaborations, and education on issues of environmental exposures and their human health effects, focusing on local interests.

*How-Ran Guo* *Zailina Hashim* *Shih-Bin Su* *Jochen Bundschuh*

